# Designing siRNA That Distinguish between Genes That Differ by a Single Nucleotide

**DOI:** 10.1371/journal.pgen.0020140

**Published:** 2006-09-08

**Authors:** Dianne S Schwarz, Hongliu Ding, Lori Kennington, Jessica T Moore, Janell Schelter, Julja Burchard, Peter S Linsley, Neil Aronin, Zuoshang Xu, Phillip D Zamore

**Affiliations:** 1 Department of Biochemistry and Molecular Pharmacology, University of Massachusetts Medical School, Worcester, Massachusetts, United States of America; 2 Department of Medicine, University of Massachusetts Medical School, Worcester, Massachusetts, United States of America; 3 Rosetta Inpharmatics, Merck and Co., Seattle, Washington, United States of America; Huntsman Cancer Institute, United States of America

## Abstract

Small interfering RNAs (siRNAs), the guides that direct RNA interference (RNAi), provide a powerful tool to reduce the expression of a single gene in human cells. Ideally, dominant, gain-of-function human diseases could be treated using siRNAs that specifically silence the mutant disease allele, while leaving expression of the wild-type allele unperturbed. Previous reports suggest that siRNAs can be designed with single nucleotide specificity, but no rational basis for the design of siRNAs with single nucleotide discrimination has been proposed. We systematically identified siRNAs that discriminate between the wild-type and mutant alleles of two disease genes: the human Cu, Zn superoxide dismutase (SOD1) gene, which contributes to the progression of hereditary amyotrophic lateral sclerosis through the gain of a toxic property, and the huntingtin (HTT) gene, which causes Huntington disease when its CAG-repeat region expands beyond approximately 35 repeats. Using cell-free RNAi reactions in *Drosophila* embryo lysate and reporter assays and microarray analysis of off-target effects in cultured human cells, we identified positions within an siRNA that are most sensitive to mismatches. We also show that purine:purine mismatches imbue an siRNA with greater discriminatory power than other types of base mismatches. siRNAs in which either a G:U wobble or a mismatch is located in the “seed” sequence, the specialized siRNA guide region responsible for target binding, displayed lower levels of selectivity than those in which the mismatch was located 3′ to the seed; this region of an siRNA is critical for target cleavage but not siRNA binding. Our data suggest that siRNAs can be designed to discriminate between the wild-type and mutant alleles of many genes that differ by just a single nucleotide.

## Synopsis

First discovered in nematodes, RNA interference (RNAi) has become an essential tool in the study of mammalian gene function. RNAi directed by small interfering RNAs (siRNAs), 21 nt, double-stranded RNAs target complementary mRNAs for destruction. siRNAs can be introduced into mammalian cells grown in culture, or even administered intravenously to rodents or primates, where they repress production of the targeted gene product. Thus, siRNA-directed RNAi has tremendous potential as a human therapeutic strategy. Dominant genetic disorders, in which a mutant allele of a gene causes disease in the presence of a second, normal copy, might be treated with therapeutic siRNAs, provided that the siRNAs could be designed to destroy the mutant, disease-causing mRNA, while leaving the normal mRNA intact. Here, Schwarz and colleagues describe an experimentally validated strategy for the design of such siRNAs. Their design strategy should facilitate the design of siRNAs targeting dominant genetic disorders such as amyotrophic lateral sclerosis and Huntington disease.

## Introduction

In the RNA interference (RNAi) pathway, small interfering RNAs (siRNAs), 21- to 23-nucleotide double-stranded RNAs, target a corresponding mRNA for post-transcriptional destruction. siRNAs act as guides for a protein complex, RISC (RNA-induced silencing complex), which mediates target RNA destruction [1–3]. Synthetic siRNAs provide a straightforward means to knock-down gene expression in vitro in cultured human cells [4] and in vivo in mice [5–8] and primates [9]. When an siRNA is complementary to its mRNA target, the siRNA directs endonucleolytic cleavage of the mRNA at a single phosphate across from nucleotides 10 and 11 of the siRNA “guide” strand, the strand loaded into the RNA-induced silencing center (RISC), triggering mRNA destruction [1,10–12]. Both strands of an siRNA can act as guides [3,10,13,14], but siRNAs are most effective when specifically designed to load the antisense strand into RISC and concomitantly destroy the sense, or “passenger,” strand [14,15]. Which siRNA strand serves as the guide reflects the relative thermodynamic stability of the 5′ ends of the two siRNA strands [14,15]. siRNAs that exhibit near absolute asymmetry, with only one strand of the siRNA capable of entry into the RISC, are said to be functionally asymmetric [14].

Some mismatches between an siRNA and its target RNA block target cleavage by RISC [13,16–22]. Thus, siRNAs can discriminate between mRNAs that differ at a single base-pair, suggesting the potential application of this mechanism to suppress mutant genes in dominant human diseases including amyotrophic lateral sclerosis (ALS) [23–26], Huntington disease (HD) [27], Alzheimer disease [28], human immunodeficiency virus infection [29,30], slow channel congenital myasthenic syndrome [31], spinocerebellar ataxia type 3 [32], sickle cell anemia [33], and cancer [34]. Because siRNAs to treat these and similar diseases would need to target single nucleotide polymorphisms (SNPs), their design is limited to the region surrounding the mutation.

ALS is an age-dependent neurodegenerative disease that can be caused by sporadic or inherited dominant point mutations in the Cu, Zn superoxide dismutase gene (SOD1) [35]. Point mutations in SOD1 have been linked to the acquisition of a toxic property by the mutant protein, rather than loss of the wild-type function of SOD1 in preventing cellular damage by destroying free oxygen radicals released from metabolic processes [36]. With many point mutations clinically identified, one possible therapy to ameliorate the symptoms of ALS would be allele-specific therapy to selectively eliminate expression of the mutant copy of the SOD1 gene. Ideally, such a therapy would target only the mutant allele, because loss of the wild-type gene in mice causes many abnormalities, including degeneration of motor neuron axons [37,38], liver cancer, and shortened life span [39], as well as reduced fertility [40,41]. HD likewise is a neurodegenerative disorder caused by a dominant, toxic gene product. HD patients suffer cell death in the cortex and striatum of the brain, causing motor and cognitive symptoms [42]. The cause of the disease is an expansion of the CAG repeat in exon 1 of the huntingtin gene (HTT), which leads to a protein containing an extended polyglutamine tract [43]. The time of onset of HD correlates with the length of the expanded CAG repeat [44,45].

To develop a general strategy for the design of single nucleotide–specific siRNAs, we examined the effect of mismatch identity and position on the ability of an siRNA to discriminate between two target RNAs that differ by only a single base. As a model system, we used siRNAs that silence the G85R point mutation in human SOD1, an allele that causes a familial form of ALS. We then tested the generality of our findings by designing siRNAs to discriminate between two alleles of HTT mRNA that differ at a SNP. Although specific-SNP isoforms are not known to correlate with HD, previous attempts to distinguish between wild-type and disease-causing HTT alleles using siRNAs targeting the disease-causing CAG-repeat expansion have proved unsuccessful [46], suggesting that allele-specific therapy may be required.

Here, we show that maximal discrimination is achieved when the siRNA:target RNA pairing is disrupted by a purine:purine mismatch; little or no discrimination is achieved by other classes of mismatches. Surprisingly, mismatches in the “seed” region of the siRNA—the six-nucleotide siRNA region that contributes the bulk of target-binding energy—do not ensure effective single nucleotide discrimination in cultured cells. In contrast, mismatches in the central and 3′ regions of the siRNA provide a high degree of single nucleotide discrimination, consistent with previous proposals that target cleavage requires that these regions pair with their target RNAs to form an A-form RNA:RNA helix [47,48]. Remarkably, a mismatch at siRNA nucleotide 16 provided more than 4-fold discrimination between two alleles for all ten siRNA:target RNA pairs examined and robust discrimination (at least 20-fold) for seven of the ten sequences tested.

## Results

### A Tiled Set of Functionally Asymmetric siRNAs Targeting Mutant SOD1

**Figure 1:**
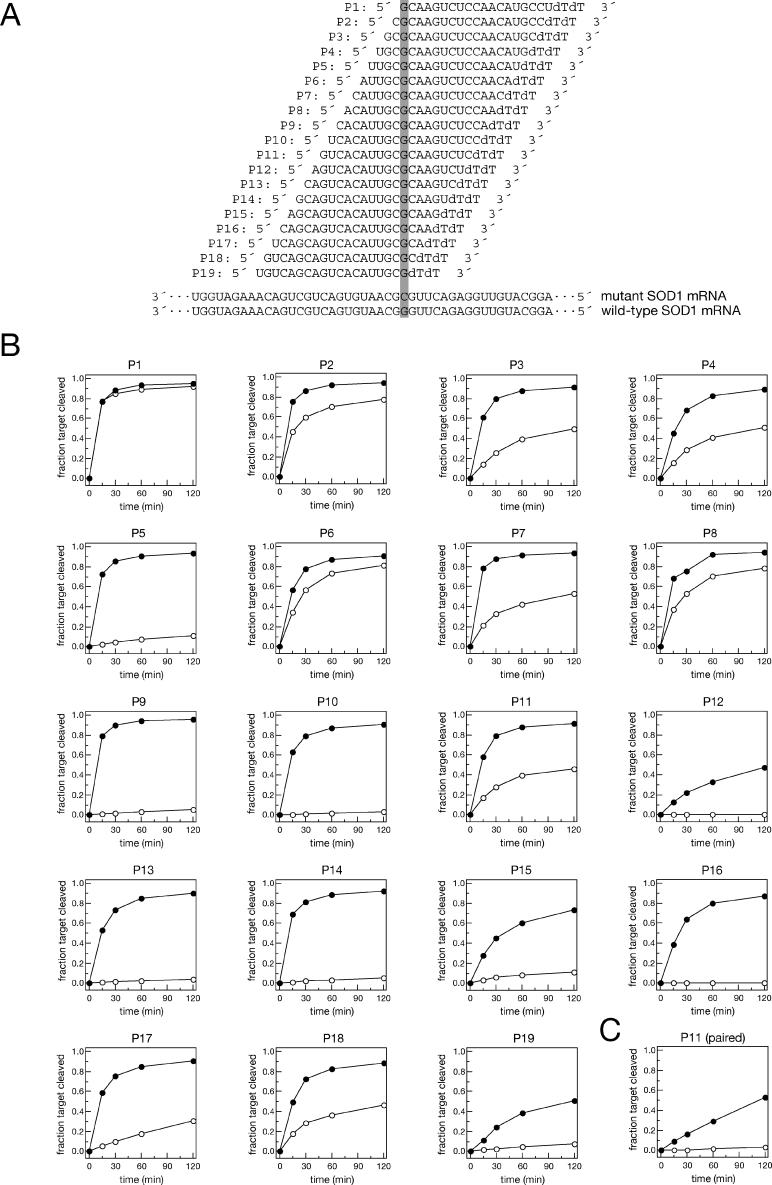
Analysis in *Drosophila* Embryo Lysate of a Tiled Set of siRNAs Targeting Human SOD1 (A) Sequences of the guide siRNA strands used, indicating the site of the G:G mismatch. The third nucleotide from the 3′ end of the sense strand of the siRNA (not shown) was mismatched with the guide, creating an unpaired 5′ end to facilitate entry of the guide strand into RISC. For example, where the first nucleotide of the guide strand was U, the sense strand was changed to C, and vice versa. Where the first guide nucleotide was G, the corresponding sense strand was designed to be A, and vice versa. The mutant (matched) and wild-type (mismatched) SOD1 mRNA sequences targeted by the siRNAs are shown below. (B) Rate of target cleavage in *Drosophila* embryo lysate of mutant (filled circle) or wild-type target (open circle) for each siRNA in the tiled set. (C) Comparison of a 5′ paired and a 5′ unpaired P11 siRNA (B) reveals that the 5′ paired siRNA exaggerates the inherent discrimination of this siRNA between matched and mismatched target RNAs.

We synthesized a set of 19 siRNAs tiling across the G85R point mutation of human SOD1 (Figure 1A). The G85R mutant contains a cytosine at position 323 of the mRNA, whereas the wild-type mRNA bears a guanosine at that position. Each siRNA fully matched the mutant SOD1 but contained a G:G mismatch with wild-type. To ensure that the antisense strand of each siRNA served as the guide in RISC, each siRNA had an unpaired, antisense-strand 5′ end, a design strategy that imparts “functional asymmetry” to an siRNA [14,15]. In vitro RNAi experiments in *Drosophila* embryo lysate demonstrated that all 19 siRNAs effectively targeted their fully matched targets, the mutant G85R allele of SOD1*,* allowing us to assess how well each siRNA discriminated against the wild-type SOD1 allele (Figure 1B). The importance of this strategy can be seen by comparing the conventionally designed—i.e., fully paired—siRNA position 11 (P11) from our previous study of allele-specific siRNAs [23] with the functionally asymmetric version used here (Figure 1B and 1C). The conventionally designed P11 siRNA showed considerable discrimination against the wild-type SOD1 allele (Figure 1C). The functionally asymmetric P11 siRNA (Figure 1B) revealed that the source of this discrimination was the poor activity of the original siRNA against the fully matched target, rather than a large difference in its activities against the two SOD1 alleles.

Analysis of the tiled set of functionally asymmetric siRNAs showed that the P5, P9, P10, P13, P14, P15, and P16 siRNAs all discriminated between G85R mutant and wild-type SOD1 (Figure 1B). Additionally, the P12 and P19 siRNAs displayed some discrimination against the mismatched wild-type target, but these two siRNAs did not show robust silencing of the perfectly matched mutant target in the cell-free RNAi reaction, consistent with the idea that an unpaired guide strand 5′ end is not the sole determinant of siRNA efficacy [15,49]. To provide a more quantitative measure of siRNA efficacy, we also determined for each siRNA in the tiled set its initial rate of cleavage in single-turnover reaction conditions (Figure S1). The initial rate of cleavage reflects the concentration of active RISC containing the antisense strand of the siRNA duplex and the inherent catalytic rate of cleavage of the targeted sequence but not the rate of product dissociation from RISC. Four siRNAs exhibited surprisingly slow initial rates of reaction: P12, P15, P16, and P19. Of these siRNAs, P12 and P19 also showed a low extent of cleavage over a longer time course (Figure 1B). In contrast, the P15 and P16 siRNAs performed well over the 2-h time course, although they showed a slow rate of initial cleavage.

### A Stringent Biochemical Test for siRNA Selectivity

**Figure 2:**
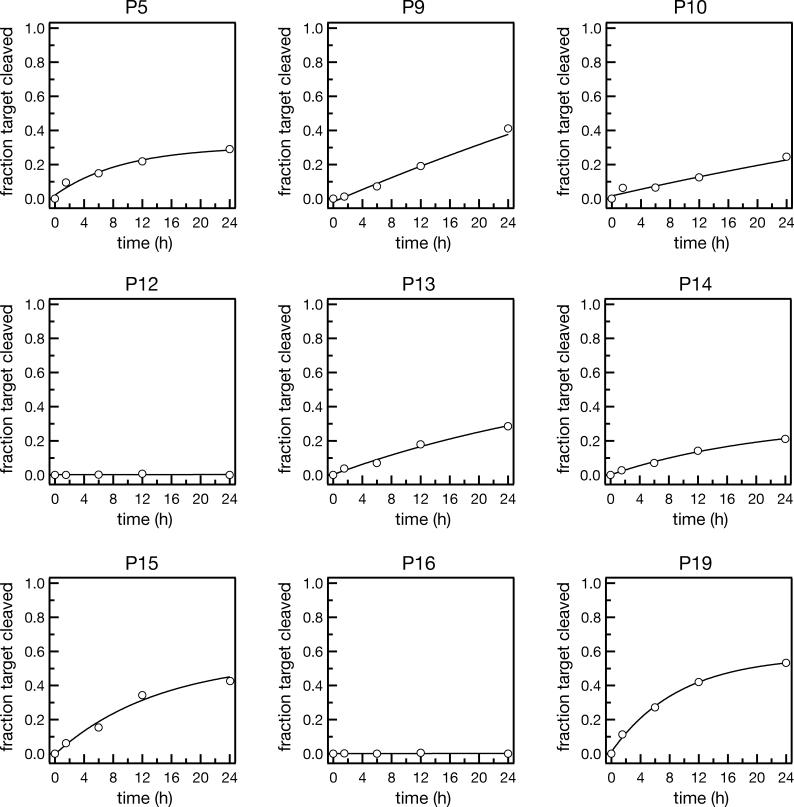
Mismatched Target Cleavage after 24-h Incubation in *Drosophila* Embryo Lysate To detect low levels of target cleavage, long-time scale reactions were performed with the concentration of RISC greater than that of the RNA target for those siRNAs (P5, P9, P10, P12, P13, P14, P15, P16, and P19) that showed high levels of discrimination between the matched and mismatched targets in Figure 1.

While some of the siRNAs in this study exhibited high levels of discrimination during a 2-h reaction, a more rigorous test of the ability of an siRNA to discriminate against a mismatched RNA target is to examine cleavage over a 24-h period. We performed 24-h cleavage reactions using a high concentration of siRNA and a low concentration of target RNA, so as to detect even a small degree of activity of the siRNA against the mismatched target (Figure 2). Under these intentionally artificial conditions, many of the siRNAs which originally showed complete discrimination against the wild-type SOD1 RNA target showed detectable levels of cleavage of the wild-type, mismatched RNA. In contrast, the P12 and P16 siRNAs showed no cleavage of the wild-type target, suggesting that the mismatch at these positions effectively blocked RISC activity, under these experimental conditions.

### Analysis of Tiled siRNAs in Cultured Human Cells

**Figure 3:**
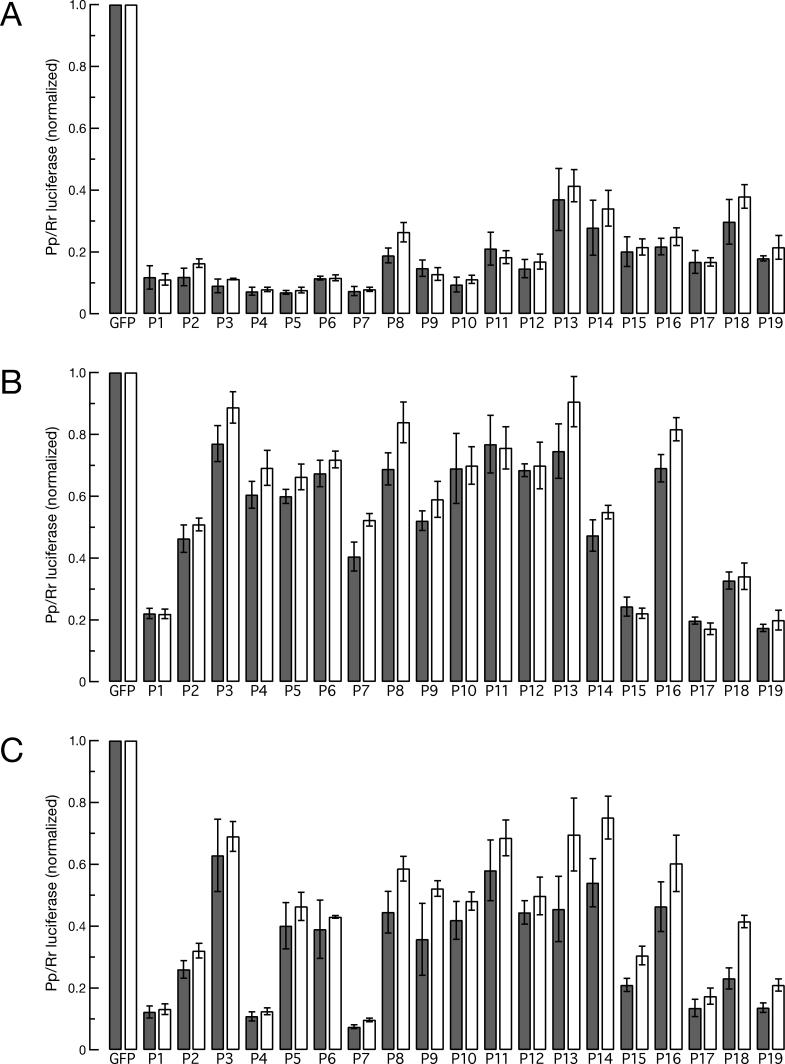
Analysis of Tiled siRNAs against an SOD1-Luciferase Fusion in Cultured Human Cells (A) Relative firefly luciferase expression for siRNAs (Figure 1A) co-transfected at either 2 nM (gray bars) or 20 nM (white bars) with a reporter plasmid containing the mutant (matched) SOD1 sequence fused to the luciferase coding sequence. (B) The same set of siRNAs was analyzed by co-transfection with a reporter plasmid containing the wild-type (mismatched) SOD1 sequence, creating a G:G clash, or (C) a reporter plasmid encoding a U at the same position, creating a G:U wobble. Each experiment was performed in triplicate and the data were normalized to luciferase expression measured using a GFP siRNA. Average ± standard deviation is shown.

To what extent does the cell-free analysis using *Drosophila* embryo lysate predict the behavior of an siRNA in a human cell? We assessed the efficacy and discriminatory power of each siRNA by co-transfecting it into HEK 293T cells with a plasmid expressing a firefly *(Photinus pyralis [Pp])* luciferase bearing either the relevant region of the wild-type or the G85R mutant SOD1 sequence cloned into its 3′-untranslated region. Silencing efficiency was determined by measuring firefly luciferase activity, relative to an untargeted *Renilla (Rr)* luciferase control, 24 h after transfection with either 2 nM or 20 nM siRNA (Figure 3). Wild-type SOD1 contains a G at position 323; in the G85R mutant, this position is a C. The siRNAs were also evaluated using a *Pp*-luciferase-SOD1 fusion target containing a uridine residue at position 323 of the SOD1 mRNA sequence. G:U wobbles were previously reported to be poorly tolerated in the seed sequence of a miRNA-like siRNA [50], suggesting that G:U mismatches in this region might provide single nucleotide specificity, although data in flies suggest that extensive pairing outside the seed can compensate for a G:U wobble pair within the seed [51].

The 19 siRNAs examined using the fully matched target RNA (the G85R mutant SOD1) silenced the reporter by at least 60%; of these, 15 silenced the reporter by 80% or more (Figure 3A). When the same set was examined using the mismatched, wild-type SOD1 reporter, ten of the 19 siRNAs effectively discriminated against the mismatched target RNA (Figure 3B). siRNAs P3, P4, P5, P6, P8, P10, P11, P12, P13, and P16 repressed wild-type reporter expression by less than 40%. Thus, most of the siRNAs that exhibited high levels of discrimination in the cell-free *Drosophila* RNAi system also discriminated in cultured human cells, including siRNAs P5, P10, P12, P13, and P16.

Next, the same set of siRNAs was co-transfected with a reporter designed to create a G:U wobble instead of a G:G mismatch. Only five siRNAs of the 19 showed effective discrimination against the wild-type SOD1 reporter (i.e., less than a 40% reduction in expression) (Figure 3C). In theory, the “seed” region of an siRNA, which mediates siRNA binding to a target RNA, should be highly sensitive to mismatches, but siRNA P3 was the only one of the six siRNAs to show more than 2-fold allele specificity when a G:U wobble was placed within the seed sequence. Rather, mismatches 3′ to the seed—siRNAs P8, P11, P13, P14, and P16—best retained the ability to discriminate against the G:U wobble. Perhaps seed mismatches are ineffective at destabilizing the binding of siRNAs bearing extensive complementarity to their targets, because base-pairs outside the seed region compensate for mismatches with the seed [51], whereas mutations 3′ to the seed disrupt the A-form helical geometry required for target cleavage [47,48].

### Off-Target Effects Reveal the Strand Specificity of siRNAs against Mutant SOD1

We tested our siRNA design strategy—unpairing the 5′ end of the antisense strand to promote its incorporation into RISC—by examining the nature of the “off-target” silencing profile of each siRNA: the constellation of mRNAs whose steady-state concentration was decreased in response to that particular siRNA. Such off-target silencing is thought to reflect seed sequence-directed binding of the siRNA to mRNAs other than its intended mRNA target [52–54]. Because the siRNA seed sequence is the primary determinant of siRNA binding, off-target mRNAs contain six-nucleotide sequences (“hexamers”) complementary to the seed sequence of the siRNA strand—sense or antisense—that directed RISC to destroy them. Thus, determining which strand gives the greatest enrichment of seed region hexamer matches to the off-target expression signatures is a measure of which siRNA strand is preferentially loaded into RISC.

**Figure 4:**
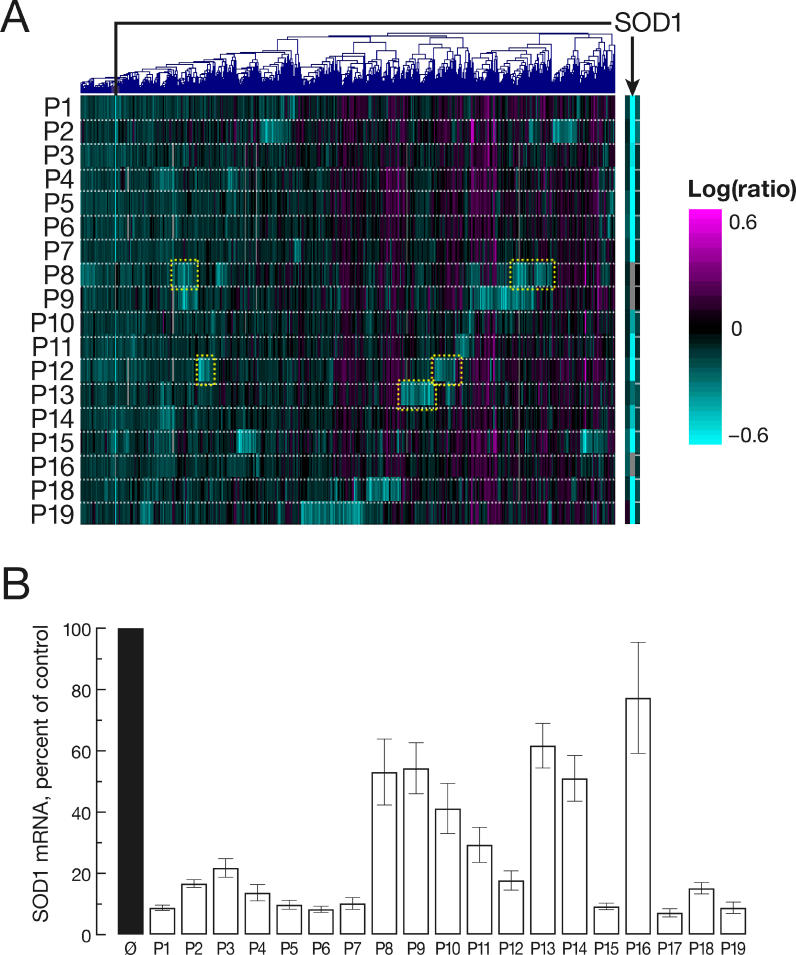
Microarray Analysis of 5′ Unpaired SOD1 siRNAs siRNAs from Figure 1A were transfected at 100 nM into HeLa cells and total cellular RNA isolated 24 h later. (A) Microarray analysis of genes down-regulated by siRNAs. Gene expression profiles were determined by competitive hybridization of amplified mRNA from siRNA-treated versus mock-treated cells. Shown is a heat map depiction of mRNAs whose expression decreased following siRNA transfection (*p* < 0.01, in one or two experiments, except for SOD1). Regulated genes are in columns, experiments in rows. The experiment using P17 was lost. Teal, genes having decreased mRNA levels compared to mock transfected cells, magenta, genes having increased mRNA levels. The color bar indicates log_10_ expression ratio transfected/mock transfected cells, −0.6 (teal) to +0.6 (magenta; i.e., 4-fold). Genes in yellow boxes are representative groups of transcripts enriched for seed region hexamer matches (see Table 1). The arrow indicates the position of the wild-type SOD1 mRNA, compared to a mock HeLa cell transfection. (B) Endogenous SOD1 mRNA levels were determined by quantitative RT-PCR for each siRNA transfection. Shown is the mean ± standard deviation of three replicate determinations for each siRNA. Endogenous wild-type SOD1 mRNA is mismatched to the siRNAs used; taller bars imply greater discrimination against the mismatched target RNA.

We transfected the siRNAs into cultured human HeLa cells at 100 nM final concentration. This siRNA concentration was selected to facilitate detection of silencing of unintended transcripts, in order to reveal the identity of the siRNA strand loaded into RISC. Total RNA was isolated from the siRNA transfected cells and analyzed by microarray transcription profiling (Figure 4A and Table 1). Each of the 18 siRNAs that we were able to evaluate contains a different seed sequence, so each should have a diagnostic off-target signature. In addition, this experiment poses a stringent test for siRNA specificity, in that (1) the transfected siRNA concentration was 5 times greater than the highest standard concentration [55] and 50 times greater than the lower effective concentration (2 nM) used in Figure 3; and (2) endogenous, wild-type SOD1 is the human mRNA most complementary to each of the siRNAs that target mutant SOD1. (Human cell lines expressing the G85R allele of SOD1 are not available.)

**Table 1:**
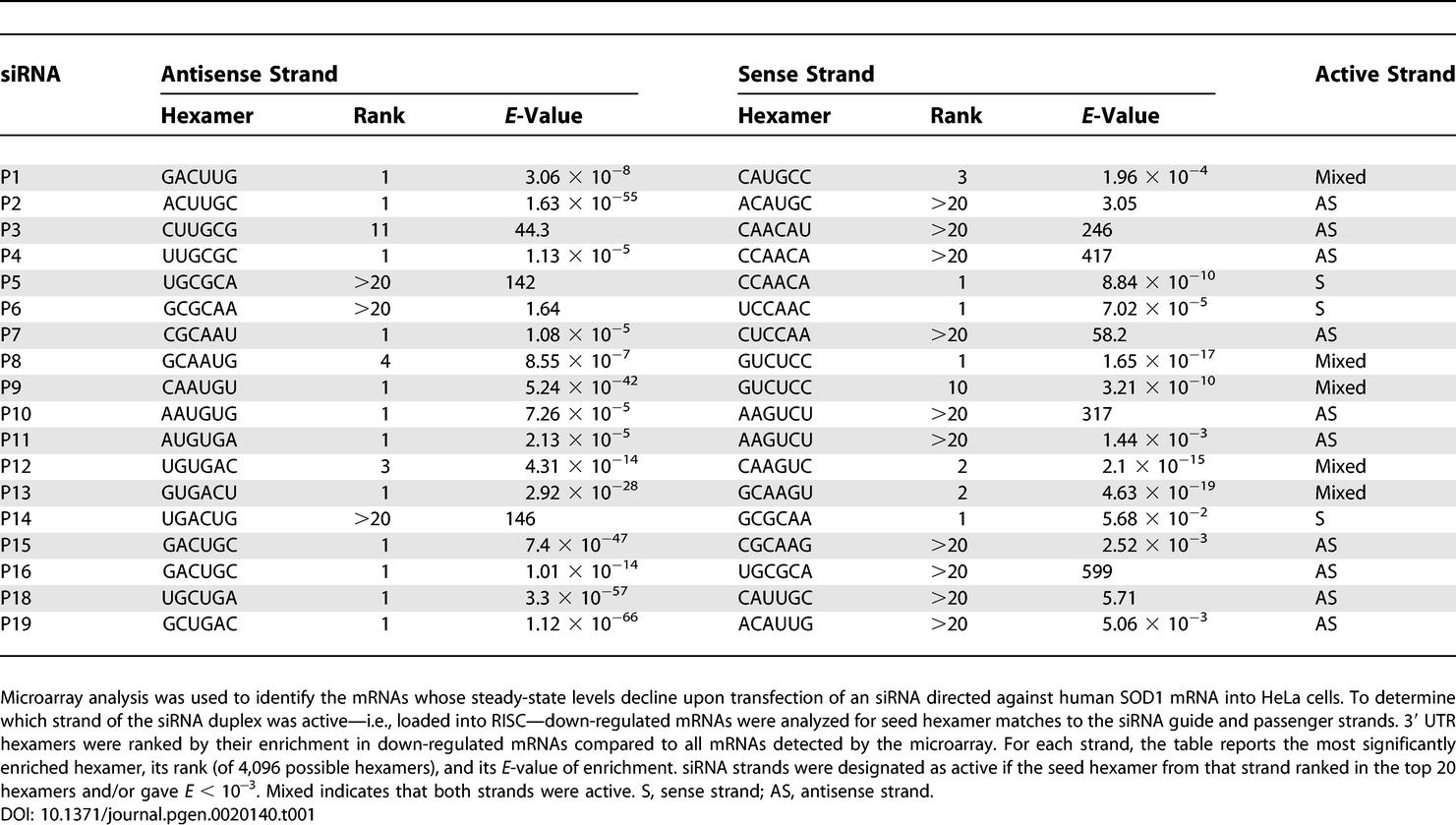
Off-Target Analysis Shows that Most of the SOD1 siRNAs Designed to Be Functionally Asymmetric Load the Guide Strand into RISC

Analysis of the off-target mRNAs down-regulated by the 18 siRNAs suggests that eight loaded predominantly their antisense strand into RISC and five loaded both strands to some degree (Table 1). siRNAs P5, P6, and P14 predominantly loaded their sense strands into RISC, suggesting that for these three siRNAs, our design strategy was not effective. Both siRNA P13, which appeared to load both strands into RISC, and siRNA P14, which loaded predominantly its sense strand, also showed little activity against their fully matched SOD1 mutant mRNA target (Figure 3A). The low activity of the antisense strands of these two siRNAs precluded further analysis of their ability to discriminate between mutant and wild-type SOD1 mRNA. The microarray data show that three siRNAs, P8, P9, and P16, triggered no detectable down-regulation of endogenous wild-type SOD1 (Figure 4A). Quantitative RT-PCR (qRT-PCR) corroborated the microarray analysis (Figure 4B). The P16 siRNA detectably incorporated only the antisense strand into RISC, whereas both the P8 and P9 siRNAs loaded both strands into RISC. The P9 and P16 siRNAs were also highly active in both *Drosophila* embryo lysate (Figures 1, 2, and S1) and HEK 293T cells (Figure 3) against the perfectly matched G85R mutant mRNA.

Notably, all of the siRNAs bearing one G:G mismatch between the siRNA seed sequence and the endogenous wild-type SOD1 gene—P2, P3, P4, P5, P6, and P7—targeted the SOD1 mRNA for destruction at this high siRNA concentration (Figure 4A and 4B). That is, none of these siRNAs retained its ability to discriminate against wild-type SOD1 when the siRNA was transfected at 100 nM. These data are consistent with the view that mismatches between the seed and its target compromise only RISC binding, not catalysis, and can therefore be overcome by increasing the concentration of the siRNA [48]. However, we cannot exclude the possibility that, at this high siRNA concentration, other small RNA-directed mechanisms account for mRNA destruction in cultured cells and that disrupting seed pairing inherently blocks Ago2-catalyzed mRNA cleavage.

### Nature of the Mismatch Determines Discrimination

**Figure 5:**
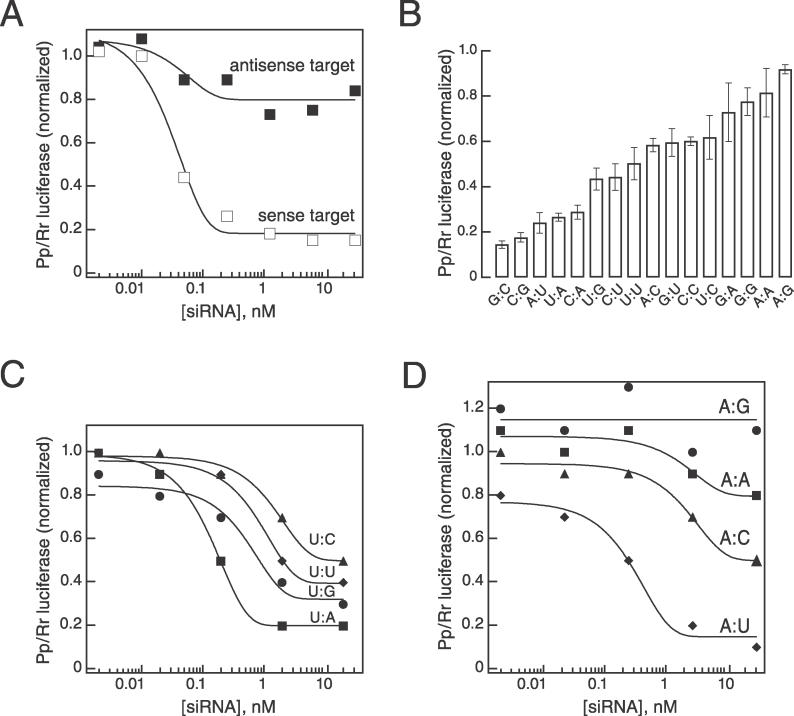
Purine:Purine Mismatches Provide the Greatest Discrimination for mRNAs Differing at a Single Nucleotide (A) The asymmetry of a fully base-paired P10 siRNA was measured using a firefly luciferase reporter containing sense or antisense SOD1 sequences. Even at high concentrations of siRNA, only the sense target (open squares) was efficiently silenced, compared to the antisense target (filled squares). Thus, the guide strand of the P10 siRNA was predominantly the antisense strand, consistent with the off-target analysis in Table 1. (B) All possible single nucleotide pairs were examined for the P10 siRNA. Among the perfectly complementary siRNA:mRNA pairs, a position 10 G:C pair triggered greater silencing than an A:U pair. Purine:pyrimidine and pyrimidine:pyrimidine mismatches displayed intermediate levels of silencing. The least silencing, i.e., greatest discrimination, was observed with purine:purine mismatches. (C) Over a range of concentrations, the pyrimidine:purine mismatches show moderate levels of discrimination compared to the perfectly matched siRNA:mRNA pair. U:C mismatches, triangles; U:U mismatches, diamonds; U:G mismatches, circles; U:A matched pair, squares. (D) Purine:purine mismatches cannot be overcome by high concentration of siRNAs and therefore the observed discrimination is not an artefact of low concentration. A:G mismatches, circles; A:A mismatches, squares; A:C mismatches, triangles; A:U matched pair, diamonds. Mismatches are reported as siRNA nucleotide:target RNA nucleotide.

Our examination of the tiled set of siRNAs targeting the G85R SOD1 mutation compared a G:C base-pair with a G:G mismatch and a G:U wobble. To extend this analysis to other types of mismatches, we synthesized four siRNAs based on the P10 siRNA sequence (Figure 1A), placing a G, C, U, or A at position 10 of the siRNA and constructed four corresponding reporter constructs expressing *Pp*-luciferase target RNAs containing each possible nucleotide across from siRNA position 10. Combining these four siRNAs with the four reporter constructs allowed us to examine all possible position 10 matches and mismatches between the siRNA and the target. The siRNA sequence used in these studies was intrinsically asymmetric, silencing a reporter complementary to the siRNA antisense strand greater than 8-fold more effectively than a reporter complementary to the siRNA sense strand (Figure 5A).

Analysis of all possible siRNA:target pairs using 2 nM siRNA concentration revealed that the strength of pairing and compatibility with an A-form RNA:RNA helix between the siRNA and its target at siRNA position 10 correlated with silencing efficacy. At least one full A-form helical turn is required for an siRNA to direct cleavage of its RNA target [47,48]. All of the perfectly matched siRNAs (G:C, C:G, A:U, and U:A) effectively silenced the reporter, with G:C and C:G pairs being the most active. Mismatches expected to be well accommodated in an A-form RNA:RNA helix (pyrimidine:pyrimidine, pyrimidine:purine, or purine:pyrimidine) displayed intermediate levels of discrimination, whereas purine:purine mismatches, expected either to destabilize the helix or to promote a stable, but nonhelical, conformation, silenced the reporter least (Figure 5B). Increasing the siRNA concentration increased the extent of silencing—i.e., decreased single nucleotide discrimination—for all siRNA:target combinations, except for the A:G mismatch, which maintained its ability to discriminate against the mismatched reporter at 20 nM siRNA (Figure 5C and 5D).

### Analysis of Purine:Purine Mismatches across the siRNA Sequence

**Figure 6:**
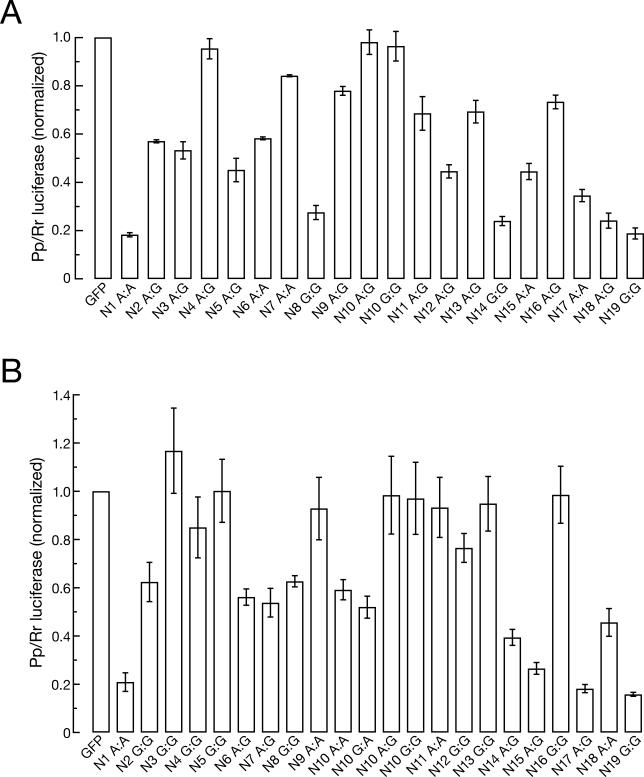
Purine:Purine Mismatches Are Tolerated at Some, but Not Other, siRNA Positions (A) Purine residues were placed at each position, N1 to N19, along the P10 siRNA. Targets were constructed so that siRNA:mRNA target pairs would result in a purine:purine mismatch. Luciferase activity was measured for each pair. (B) The P4 siRNA (Figure 1A) was used as a scaffold for the analysis of the effect of purine:purine mismatches on reporter silencing. Taller bars correspond to greater single nucleotide discrimination.

Our data suggest that purine:purine mismatches provide the highest level of discrimination against mismatched targets. To corroborate these findings, we examined the effect of a purine:purine mismatch at the 19 positions (N1 to N19) of a single siRNA sequence, the P10 siRNA. For each purine position in the P10 siRNA, a reporter was constructed that expressed a *Pp* luciferase mRNA with a purine at the corresponding target position. For pyrimidine positions in the P10 siRNA, a variant siRNA was synthesized substituting a single pyrimidine with a purine so as to create a purine:purine clash with the reporter mRNA. Seven siRNAs reduced expression of the mismatched reporter to less than 40% of the unsilenced level: N4, N7, N9, N10, N11, N13, and N16 (Figure 6A).

The same method of analysis was applied to the P4 siRNA (Figure 6B). Luciferase silencing was disrupted the least by siRNA:target mRNA combinations that placed a single purine:purine mismatch at siRNA guide position 3, 4, 5, 9, 10, 11, 12, 13, or 16. Intriguingly, seed sequence mismatches—at positions 3, 4, and 5—were strongly discriminatory for this siRNA, which has the most thermodynamically stable seed sequence pairing of all the siRNAs in this study.

For the P4 siRNA scaffold, G:G and A:G mismatches at position 10 were more selective than A:A or G:A mismatches. While single nucleotide base-pairs can either stabilize or destabilize a helix, depending on the identity of both the mismatch and the adjacent base-pairs [56], the effect of G:G, A:G, A:A, and G:A mismatches flanked by U:A base-pairs has not been experimentally determined.

### Position 16 Mismatches Generally Discriminate Well

Throughout our analyses—including cell-free RNAi reactions, reporter transfections, and microarray and quantitative-PCR analysis of endogenous mRNA—purine:purine mismatches at siRNA position 16 consistently discriminated against the mismatched target. We therefore sought to test the generality of an siRNA position 16 mismatch as a strategy for designing allele-specific siRNA. We synthesized ten distinct siRNA-mRNA pairs bearing purine:purine mismatches: five targeting an SOD1 point mutation and five targeting an HTT SNP. For comparison, a fully matched siRNA was synthesized for each target. Each pair of mismatched and matched siRNAs targeted a site inserted into the 3′ untranslated region of *Renilla* or firefly luciferase (Table 2). Reporter silencing, relative to a cotransfected luciferase control, was determined for each siRNA over a concentration range from 0.001 nM to 20 nM. The siRNA concentration producing half-maximal silencing (IC_50_) was calculated for the match or mismatched siRNAs (Table 2).

**Table 2:**
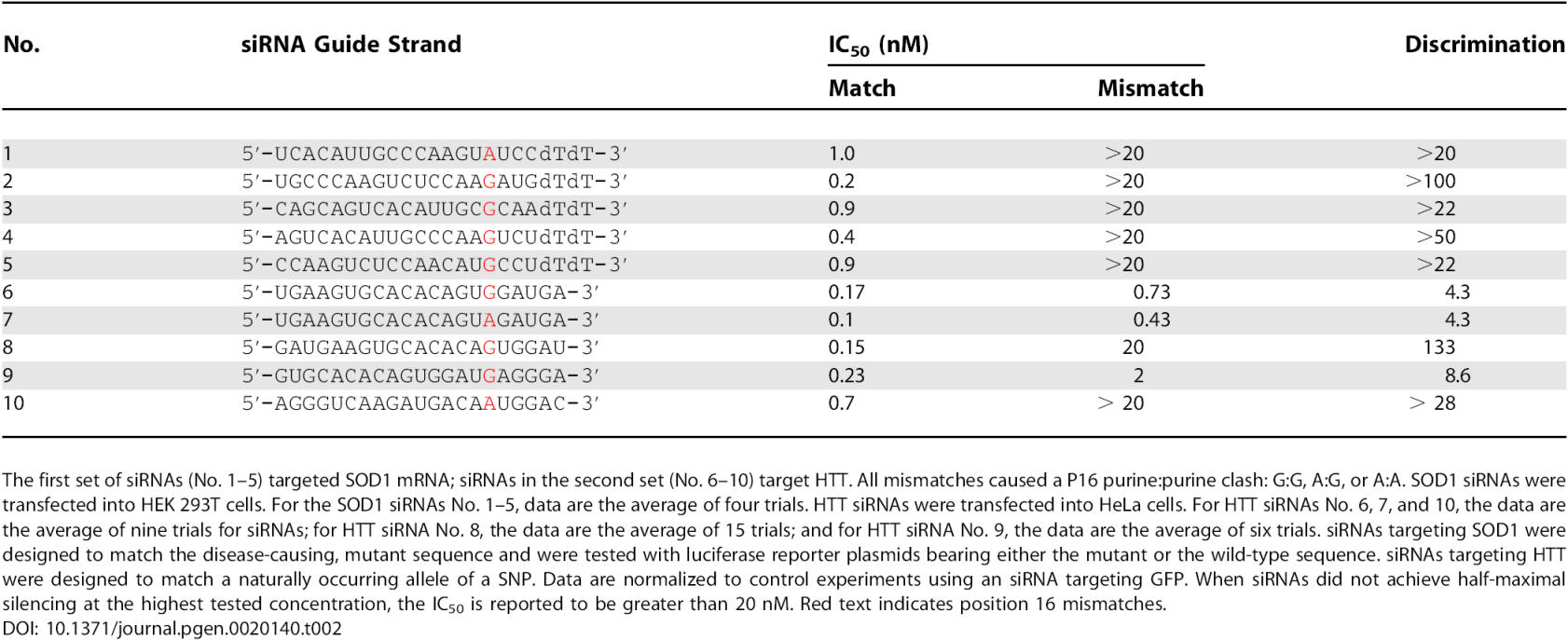
Mismatches at Position 16 Confer a High Level of Single Nucleotide Discrimination

For all ten siRNA pairs tested, the IC_50_ for the siRNA:target combination with the position 16 mismatch was greater than that for the fully matched. For seven of the ten siRNA pairs, the IC_50_ was at least 20-fold greater for the mismatched siRNA:target combination.

## Discussion

The use of single nucleotide–specific siRNA to reduce expression of mutant, disease-causing alleles holds promise for the treatment of dominantly inherited human diseases caused by point mutations that lead to a gain of function. Moreover, human disease alleles often differ from their wild-type counterparts by SNPs that do not themselves cause the disease phenotype. By targeting the SNP isoform present in the disease allele, single nucleotide–specific siRNA might be used to reduce selectively expression of the disease-causing allele without altering expression of the wild-type allele. It is therefore critical to understand the parameters for designing siRNAs with optimal discrimination between mRNAs that differ at a single nucleotide. Previous studies hinted that purine:purine mismatches between an siRNA and its target mRNA provide the greatest level of discrimination [23,33,57], probably reflecting greater disruption of the central A-form helix between the siRNA and mRNA that is required for siRNA-directed cleavage of the mRNA target [47,48]. Our systematic analysis here demonstrates the generality of this principle. Therefore, a point mutation or SNP in which a purine is changed to a pyrimidine increases the probability that an siRNA can be designed to discriminate between two alleles.

Because the last two nucleotides of an siRNA appear not to contribute to binding specificity [20] and may in fact slow RISC-catalyzed target cleavage by slowing the release of products [48], there are 19 siRNAs that, in theory, could be specific for any given point mutation. Here, we show that the extent of single nucleotide discrimination varies considerably among the 19 different positions. Using a combination of cell-free RNAi reactions and cultured human cell transfection experiments, we found that placing a purine:purine mismatch at specific siRNA positions, particularly P10 and P16, predisposes the siRNA to discriminate effectively between two alleles that differ at a single nucleotide. P5, P9, P10, P12, P13, and P16 mismatches consistently discriminated between two alleles, as tested with three different experimental strategies. Cell-free RNAi reactions using *Drosophila* embryo lysate provided an initial screen for candidate siRNAs capable of distinguishing between target alleles. Subsequent studies in cultured human cells assessed these siRNAs for both potency against the matched target and effective discrimination against the mismatched target (Figure 3), as well as determining siRNA strand choice by off-target profiling (Figure 4). In general, mismatches in the 5′ seed region of the siRNA, which is responsible for binding and recognition [48,50,53,54,58–60], provided only moderate single nucleotide discrimination, which was overwhelmed by high siRNA concentrations. In contrast, mismatches 3′ to the seed sequence promoted robust single nucleotide discrimination. This discrimination was most apparent for mismatches at positions 10 and 16, but also P9, P12, P13, and P14. In fact, in each assay used here, a mismatch at siRNA guide position 16 consistently showed high levels of discrimination against a mismatched RNA target, even at 100 nM siRNA concentration. Intriguingly, the function of plant miRNAs, which direct siRNA-like cleavage of their mRNA targets, also appears to be sensitive to position 16 mismatches between the small RNA and target: no position 16 mismatches and few G:U wobble pairs have been identified between plant miRNAs and their experimentally validated mRNA targets [61]. Thus, the sixteenth nucleotide of small RNA guides in both plants and animals may play a biochemically distinct role in directing target RNA cleavage.

A clear implication of our results is that siRNAs best discriminate between alleles when the conformation of the siRNA with the mismatch target acts to block catalysis, rather than binding, and is therefore less sensitive to siRNA concentration. Although we do not understand the detailed biochemical basis for this effect, our data suggest that the conformation of the helix formed between the small RNA and its target is monitored by some protein factor—perhaps even the core RISC protein Argonaute2 itself, the siRNA-directed RNA endonuclease that destroys the target mRNA.

## Materials and Methods

### General methods.

Preparation of *Drosophila* embryo lysate and target RNAs, siRNA annealing, and in vitro RNAi reactions were as described [12,62,63]. SOD1 mutant and wild-type RNAs were transcribed from BamHI-linearized plasmids [64] with recombinant histidine-tagged T7 RNA polymerase. Approximately 5 nM target RNA and 50 nM siRNA were used in Figure 1; approximately 0.5 nM target and 100 nM siRNA were used in Figure S1. Gels were dried and exposed to PhosphorImager (Fuji, Tokyo, Japan) plates, analyzed using an FLA-5000 PhosphorImager, analyzed, and quantified using ImageGuage 3.45 (Fuji), Excel X (Microsoft, Redmond, Washington, United States), and Igor Pro 5.01 (Wavemetrics, Lake Oswego, Oregon, United States).

### Cell culture, transfection, and luciferase assays.

HeLa cells were propagated and maintained as described [65]. HEK 293T cells were maintained in Dulbecco's modified Eagle medium (Invitrogen, Carlsbad, California, United States), supplemented with 10% fetal bovine serum, 100 U/ml penicillin, and 100 μg/ml streptomycin. HTT sequences were engineered into the 3′ UTR of the pRLTK *Renilla* luciferase vector (Promega, Madison, Wisconsin, United States) using 55-bp DNA oligonucleotides (IDT, Coralville, Iowa, United States) designed to create 5′ overhangs when annealed, allowing their insertion into the plasmid XbaI site. Plasmid constructs were verified by bidirectional sequencing. SOD1 sequences were cloned into the 3′ UTR of the firefly luciferase mRNA (pGL2 control, Promega) into NdeI and SpeI sites engineered into the plasmid by annealing two 39-nucleotide DNA oligos and ligating them into the vector. Transfections were carried out using LipofectAMINE 2000 (Invitrogen) in 24-well plates using 0.25 μg of pGL2 firefly luciferase (Promega) and 0.1 μg of *Renilla*-HTT constructs or in 96-well plates using 2 μg/ml firefly fusion vector and 0.1 μg/ml *Renilla* vector. Cells were washed in 1× PBS (Invitrogen) and harvested 24 h after transfection in 1× passive lysis buffer (Promega). Luciferase levels were determined using the Dual Luciferase kit (Promega) and a Veritas Microplate Luminometer (Turner Biosystems, Sunnyvale, California, United States). *Renilla* luciferase/firefly luciferase ratios were normalized to a transfection with a GFP siRNA (Qiagen, Valencia, California, United States). IC_50_ values were determined by fitting the data to the Hill equation with *n* = 1. For siRNAs in which the half-maximal concentration for silencing was not reached at the highest concentration tested, IC_50_ values were reported as greater than the highest concentration tested.

### Microarray and quantitative PCR analysis.

HeLa cells were from the American Type Culture Collection (Rockville, Maryland, United States). Cells were plated 24 h prior to transfection with OligofectAMINE (Invitrogen). Duplexes were used at a final concentration of 100 nM. Cells were transfected in six-well plates and RNA was isolated 24 h following transfection. Total RNA was purified using the RNeasy kit (Qiagen). Microarray analysis was performed as described [53,66,67]. Amplified cRNA from siRNA-transfected cells was hybridized against cRNA from mock-transfected cells (treated with transfection reagent in the absence of RNA duplex). Ratio hybridizations were performed with fluorescent label reversal to eliminate dye bias. Error models have been described previously [53,66,67]. Data were analyzed using Rosetta Resolver (Rosetta Biosoftware, Seattle, Washington, United States). mRNA levels were also measured by qRT-PCR using an ABI PRISM 7900HT Sequence Detection System and Assays-on-Demand gene expression products (Applied Biosystems, Foster City, California, United States). SOD1 mRNA was measured using ABI assay No. Hs00533490_A1 and normalized to β-glucuronidase mRNA, measured using ABI assay No. 4310888E.

## Supporting Information

Figure S1 Initial Rates for siRNA-Mediated Cleavage in *Drosophila* Embryo Lysate Reactions were performed using an excess of RISC over substrate RNA in order to ensure single-turnover conditions. The initial rate of reaction was determined using data from the first 120 s. Filled circles, mutant siRNA with mutant (matched) target; open circles, mutant siRNA with wild-type (mismatched) target.
(401 KB PDF)

### Accession Numbers

Unprocessed microarray data have been deposited in the NCBI Gene Expression Omnibus database (http://www.ncbi.nlm.nih.gov/geo) under the accession number GSE5291.

## Abbreviations

ALS: amyotrophic lateral sclerosis
RISC: RNA-induced silencing complex
RNAi: RNA interference
siRNA: small interfering RNA
SNP: single nucleotide polymorphism
