# Supplementary material for: Designing siRNA That Distinguish between Genes That Differ by a Single Nucleotide
Source: PLoS Genet. 2006 Sep 8;2(9):e140. doi: 10.1371/journal.pgen.0020140 (PMC1560399; doi:10.1371/journal.pgen.0020140)

Schwarz et al., Supplemental Figure 1

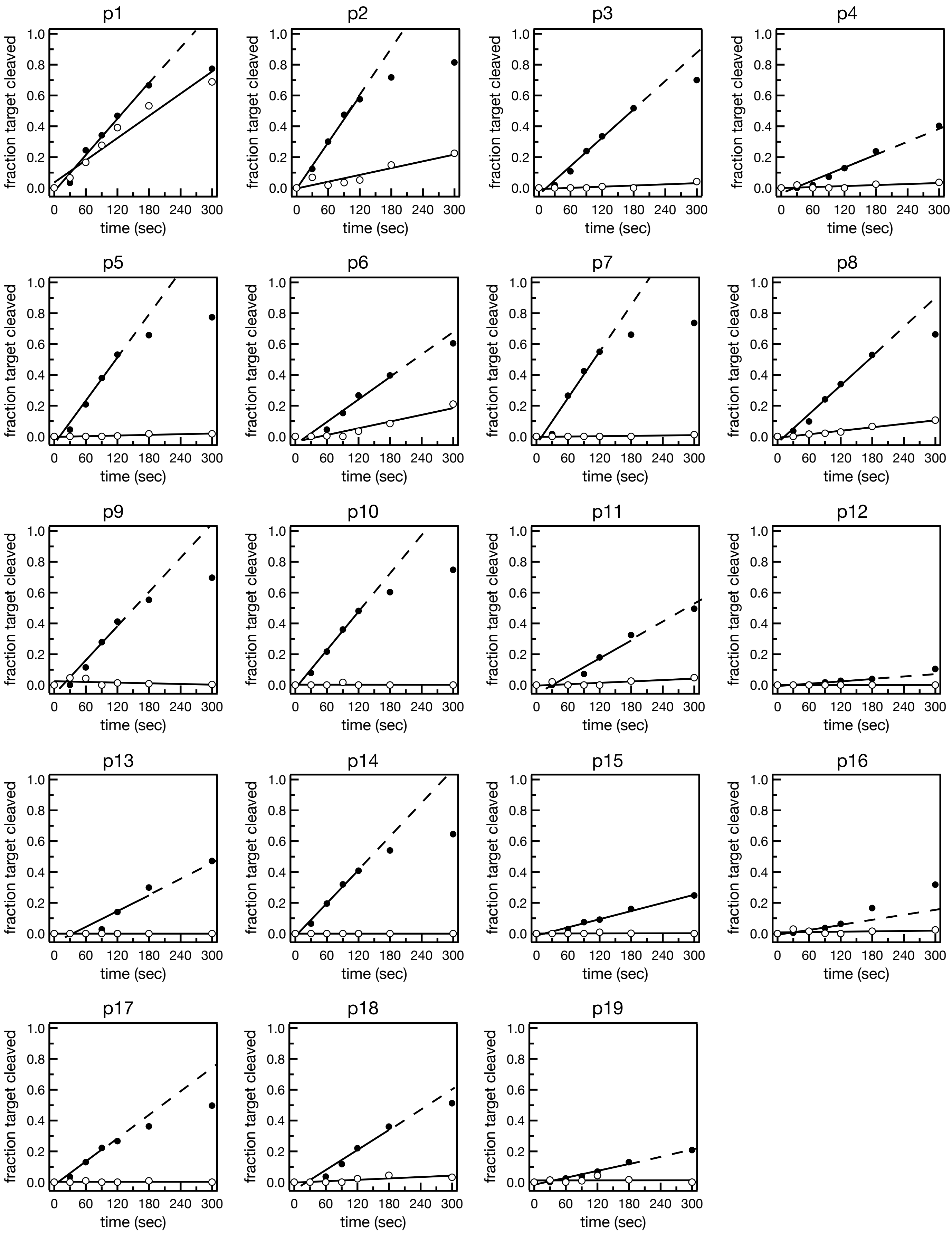

Supplement: Supplementary file 1 [file pgen.0020140.sg001.pdf]
